# Cornual pregnancy rupture and massive hemorrhage: A case report

**DOI:** 10.1097/MD.0000000000036383

**Published:** 2023-12-01

**Authors:** Xiaqin Cai, Yizhou Fu, Ke Hong, Yuefang Zhou, Guangju Qi

**Affiliations:** a Department of Obstetrics and Gynecology, Tongde Hospital of Zhejiang Province (Zhijiang Branch District), Hangzhou, China.

**Keywords:** case report, cornual pregnancy, diagnosis, treatment

## Abstract

**Background:**

Corneal pregnancy is rare and difficult to detect in the early stages. Due to the abundant blood supply in this area, a rupture can result in massive internal bleeding, shock, and even death. Therefore, immediate surgery is necessary, and patients must replenish their blood volume as soon as possible to ensure blood supply to important organs. For those whose blood pressure cannot immediately rise, surgery should be performed while resisting shock to buy time.

**Case summary:**

We present the case of a 34-year-old Chinese woman at 19 weeks of gestation who had a corneal pregnancy. No abnormalities were detected in the examinations in the first trimester. This patient was 19 weeks pregnant and sought medical advice due to sudden lower abdominal pain, syncope, and hemorrhagic shock. After rescue and treatment, she recovered and was discharged from the hospital, afterwards, the patient gave birth to a child 7 years later.

**Conclusion:**

The early diagnosis of cornual pregnancy is mainly based on ultrasound. However, there is a high incidence of missed diagnosis and misdiagnosis of this disease. Patients may face serious and life-threatening conditions in case of the rupture of cornual pregnancy. This disease can be mainly treated by surgery.

## 1. Introduction

Cornual pregnancy is a kind of ectopic pregnancy in which the fertilized egg is implanted in the uterine horn at the junction of the uterus and the opening of the fallopian tube. Patients with corneal pregnancy account for only 2% to 3% of all cases of ectopic pregnancy.^[1]^ However, the mortality of patients with corneal pregnancy is significantly higher than that with other ectopic pregnancy types.^[2]^ If an abnormal implantation position is detected at an early stage, the pregnancy may be terminated. Due to the special implantation site, atypical clinical manifestations, and late appearance of symptoms and signs of corneal pregnancy, early diagnosis is difficult, and missed diagnosis and misdiagnosis occur frequently. Due to the rich blood supply of the uterine horn, uterus ruptures may cause massive hemorrhages in the abdominal cavity. If the rescue is not timely, the uterus may be removed or the life of the patient may be endangered. Therefore, it is necessary to perform an early and accurate diagnosis. In this study, the clinical data of 1 patient with cornual pregnancy, who delivered a child 7 years later, were systematically reviewed. These findings are expected to provide new insights into the diagnosis and treatment of this disease in clinical practice.

The Tongde Hospital of Zhejiang Province Ethics Commission for human research sanctioned the study, ensuring the ethical standards and integrity of the research were maintained, and all patients gave informed consent.

## 2. Case presentation

### 2.1. Clinical presentation

A 34-year-old woman presented to the emergency department with a complaint of menstrual arrest for 19W, abdominal pain for over 4 hours, accompanied by syncope once.

The patient had regular menstruation in the previous, and the last menstruation occurred on April 2, 2014, the same amount as the normal menstruation. She felt fetal movement from the fourth month after pregnancy. She received regular examinations at the local maternal and child health care hospital. At 4 o’clock that morning, she suddenly felt persistent and severe abdominal pain. There was no obvious feeling of anal distension, nausea, and vomiting. Besides, no fever, chills, and vaginal bleeding were reported. At 4:00 am on August 15, 2014, she suffered from lower abdominal pain, then fainted once, and fell on the ground. Hence, she came to the hospital for emergency treatment at 8:25 on August 15, 2014. She presented a pale face and a poor mental state, with cold limbs and a faint pulse.

This patient had a history of laparoscopic surgery due to “ectopic pregnancy” in 2011, and a history of pelvic inflammatory diseases. Moreover, she suffered from atrophy of the left kidney due to kidney stones. Based on these findings, 2 routes of venous channels were opened immediately, and infusion was used for anti-shock treatment (including 1000 mL of crystal and 500 mL of colloid). Subsequently, blood preparation was actively carried out. Then, the rescue plan was performed, and the pregnant and lying-in women rescue team was notified at the same time. After the critical condition was explained to the patient and her family members, they signed the informed consent form and received the critical notice.

The patient had no specific personal and family history. At the initial inspection, the patient had a blood pressure of 62/32 mm Hg and a pulse rate of 100 beats per minute. The patient is pale face, cold limbs, and slight shortness of breath. Then, this patient was transferred to the ward, with a heart rate of 100 times/min, abdominal distension such as the month of pregnancy, 2 fingers under the umbilicus at the bottom of the uterus, not hard, full abdominal tenderness, marked by tenderness under the umbilicus and the right lower abdomen, obvious rebound pain, not reaching uterine contraction, fetal heart sound 148 times/min. The liver and spleen were not touched under the ribs, turbidity was removed (+), and no edema was found in both lower limbs. The gynecologic examination revealed the following results: there was no vaginal bleeding and no internal diagnosis.

The emergency blood routine examination revealed the following results: white blood cell count: 18.78 * 10 ^ 9/L, neutrophil percentage: 89.2%, hemoglobin: 82 g/L, hematocrit: 0.26, platelet: 166 * 10 ^ 9/L, and hypersensitive C-reactive protein: 2.9 mg/L. Abdominal puncture extraction did not coagulate. The emergency B-ultrasound examination showed that the fetal heart sound was 155 times/min. The placenta was attached to the right wall of the posterior wall of the uterus. The echo of the basement membrane of the placenta was continuous. No obvious liquid dark area was detected in the rear. The echo of the right wall near the uterine corner was uneven. A few linear liquid dark areas appeared to be connected with the right abdominal cavity. Large areas of the hyperechoic omentum were observed in the right abdominal cavity. Free liquid dark areas were observed in the liver, spleen, renal recess, and the perihepatic, pelvic, and abdominal cavity, with sound transmission. These findings revealed a single live fetus, approximately 19 + W. A large volume of ascites suggested that there may be ruptures of pregnancy in the right uterine horn. The final diagnosis of the case is cornual pregnancy rupture.

### 2.2. Interventional procedure

After admission, relevant rescue measures were adopted to immediately improve the liver and kidney functions. Then, blood coagulation and other tests were conducted, and 1000 mL of red blood cells were prepared. During emergency exploratory laparotomy, it was found that the uterus was enlarged, presenting with 4 + months of pregnancy, and the right uterine horn was ruptured with a rupture size being about 3 * 3 cm. The placenta tissue and amniotic sac protruded from the rupture, with high tension, without obvious active bleeding. The size of the placenta was about 5 * 5 * 5 cm, and that of the amniotic sac drum was about 6 * 6 * 6 cm. There was no abnormality in the appearance of the right fallopian tube, the left fallopian tube, and the bilateral ovaries. During blood aspiration and exploration in the abdominal cavity, it was found that the amniotic sac was gradually enlarged and the fetus was delivered from the right corner of the uterus. After the amniotic sac was punctured, the fetus was delivered, and oxytocin 20 IU was injected intramuscularly into the body of the uterus. It was found that part of the placenta was delivered spontaneously, and part of the placenta adhered to the corner of the uterus. The adherent placenta was stripped by hands. After relevant examinations, the right uterine horn was repaired as per the following steps. Firstly, 1 # intestinal sutures were used to intermittently suture the rupture of the right uterine horn, and the uterine contraction was unfavorable during surgery. Subsequently, 20 IU of oxytocin was administered intravenously, 10 IU of Murphy tube was administered, and a total of 750 μg of Xinmupei was injected into the uterine body. Then, 1 # absorbable intestinal sutures (B-Lynch sutures) were used to suture the anterior and posterior walls of the uterus. One drainage tube was placed in the abdominal wall and the pelvic cavity, respectively. The intraoperative infusion was 6000 mL, the intraoperative bleeding was about 5000 mL, the blood transfusion was 1000 mL, and the plasma was 600 mL. The urine was clear with a volume of 640 mL. After surgery, the patient was transferred to the intensive care unit. The uterine contraction, anti-infection, fluid replacement, blood transfusion, and symptomatic support treatment were performed routinely. This patient recovered and was discharged from the hospital 11 days after surgery. In 2021, she was pregnant again and delivered a child.

### 2.3. Outcome and patient perspective

Postoperatively, neutrophil granulocytes returned to normal levels. The patient delivered a live, healthy, full-term babyviaa cesarean section. After 2 years of follow-up, the patient and baby were found to be healthy.

## 3. Discussion

Cornual pregnancy is a rare ectopic pregnancy caused by repeated abortion, repeated curettage and other uterine cavity operations. In recent years, the prevalence of salpingitis, endometritis, and uterine cavity adhesion is increasing with the rapid development of assisted reproductive technology, resulting in a higher incidence of cornual pregnancy. Most patients may terminate the pregnancy due to the abnormal implantation position at an early stage, and it is rare to reach full term. Due to the special anatomical factors, there is a large blood supply in the uterine horn, and the decidua is underdeveloped. The risk of such pregnancy-related complications as uterine ruptures and placental implantation is easy to occur under the erosion of the placental villi. Patients may suffer from cornual pregnancy ruptures in the middle pregnancy period (after 12 weeks). The massive hemorrhage in the abdominal cavity induced by cornual pregnancy ruptures can endanger the lives of these patients. Therefore, it is of great significance to improve the diagnosis and emergency treatment of cornual pregnancy.

At present, Janson and other diagnostic criteria are used in clinical practice, including abdominal pain, vaginal bleeding with asymmetric enlargement of the uterus, followed by abortion or vaginal delivery; unilateral enlargement of the uterus with a lateral displacement of the round ligament under a direct vision; the presence of the placenta in the uterine horn. Patients who met any of the above criteria can be diagnosed with cornual pregnancy. In early pregnancy, the detection rate of cornual pregnancy was about 82.9%.^[3]^ The typical manifestation of this disease is that the pregnancy sac is an enlarged uterine horn on 1 side, surrounded by complete basal and interstitial line signs, namely that the endometrial line extends to the uterine horn and reaches the edge of the pregnancy sac.^[4]^ Carius BM et al^[5]^ proposed the ultrasound diagnostic criteria for cornual pregnancy. Specifically, there was no pregnancy sac in the uterine cavity. The pregnancy sac was located in the swollen cornual and separated from the uterine cavity. The periphery of the pregnancy sac was a thin wall base with a thickness of <0.5 cm. The pregnancy mass was close to or adjacent to the endometrium.

From the perspective of medical history, ultrasound examinations in early pregnancy may not indicate the abnormal position of the pregnancy sac. The possible reason is that the implantation position of the pregnancy sac is inclined to 1 side of the uterine horn, but grows towards the direction of the uterine cavity. The abnormal position of the pregnancy sac during ultrasound examinations is not typical, which will arise the vigilance of the ultrasound doctor. The missed diagnosis and misdiagnosis occur frequently. Similarly, only the placental attachment is located in the uterine horn, while the fetus grows into the uterine cavity, which can maintain the cornual pregnancy in middle and late pregnancy. Hence, it is difficult to identify the typical manifestation of asymmetric enlargement of the uterus by ultrasound examinations.^[4]^ It has been reported in China that 1 patient with 33-week pregnancy suffered from lower abdominal pain and was misdiagnosed as “appendicitis.” Besides, it was found that there was a massive hemorrhage in the abdominal cavity due to the rupture of cornual pregnancy during laparotomy. Some scholars have proposed that “the cervix is longer and the lower segment of the uterus is thickened” or “the amniotic cavity is far from the cervix.” These findings, as well as the placenta implantation at 1 corner of the uterus, constitute important clues for the diagnosis of cornual pregnancy in the second and third trimester of pregnancy.^[6]^ Therefore, it is considered that the long axis section of the cervix should be examined by ultrasound for pregnant patients at any gestational week. In addition, this examination point can also be considered in cases that cannot be determined by ultrasound. Magnetic resonance imaging (MRI) can be employed to more accurately determine the position of the placenta attached to the uterine cavity and identify whether it is associated with implantation and other abnormalities. However, the application of MRI is restricted due to its high costs.^[7]^

Due to the different outcomes of patients with cornual pregnancy, there are diverse and personalized treatment methods for these patients. If a patient with cornual pregnancy is found in early pregnancy, the pregnancy would be terminated immediately. She can be treated by hysteroscopic curettage, laparoscopic or laparotomy cornual wedge resection, medical abortion, B-ultrasound positioning curettage, and uterine cavity observation and suction system curettage. However, it is difficult to perform early diagnosis and treatment for cornual pregnancy due to its abnormal implantation site, atypical clinical manifestations, and late appearance of symptoms and signs. The rupture of cornual pregnancy can cause a massive hemorrhage in the abdominal cavity of the patient, and even endanger her life. Emergency laparotomy is the only option to save their lives. Therefore, it is very important to make a clear diagnosis and select a reasonable treatment plan at the early or middle and late stages. Accurate ultrasound diagnosis is the premise of selecting a reasonable treatment plan. A treatment method with a small trauma, fast recovery, and favorable prognosis can be selected according to the physical condition, the pregnancy attachment site, the size of the mass, and the fertility requirements of patients.

In recent years, with the wide application of vaginal ultrasound examinations, most patients with cornual pregnancy can be diagnosed in early pregnancy. They can receive drug treatment at an early stage, including mifepristone combined with misoprostol and systemic methotrexate (MTX). It has been reported that the success rate of systemic application of MTX is 80%.^[7]^ However, MTX may induce significant side effects, which would cause damage to liver and kidney functions, leukopenia, thrombocytopenia, gastrointestinal ulcer, etc. During the abortion and curettage for patients with cornual pregnancy, due to its special anatomical structures, the difficulty of surgery is significantly increased. Insufficient preparation before surgery may cause uterine perforation or aspiration leakage. It is necessary to locate the uterus with B-ultrasound, laparoscope exploration, and curettage, which contributes to correct judgment and timely treatment. Besides, this examination is suitable for patients at an early stage who have small and unbroken packages and require the preservation of reproductive function. For patients who have a history of cornual pregnancy surgery, ultrasound during pregnancy may indicate an abnormal fetal position or high risks of the placenta near cornual pregnancy. Based on that, monitoring should be strengthened during pregnancy, especially ultrasound examinations. Further, the possibility of cornual pregnancy and related risks should be explained to the patient. If necessary, MRI should be performed to assist in evaluating the situation of the placenta. In case of unexplained abdominal pain, the medical history or pregnancy examination results should be comprehensively reviewed. Moreover, more attention should be paid to the obstetric complications of severe rupture of cornual pregnancy. If there is a progressive decrease in hemoglobin or an increase in abdominal effusion indicated by imaging, exploratory laparotomy should be performed in time.

This patient had a history of pelvic inflammatory diseases, ectopic pregnancy, and laparoscopic surgery, which may be the main cause of cornual pregnancy. She received ultrasound examinations before seeking medical advice in our hospital. The abdominal ultrasound examination alone in early pregnancy may cause a deviation from the real position of the pregnancy sac, thereby inducing failure to detect cornual pregnancy in early time. Therefore, vaginal ultrasound examinations in early pregnancy are of great significance to cornual pregnancy. At the same time, the normal HCG elevation results and the absence of vaginal bleeding in this patient also affected the early diagnosis. The patient fainted once before admission, and her face turned pale after admission, with decreased blood pressure. The B-ultrasound results showed that the gestational age of the single live fetus was 19 + W. A large volume of ascites suggested that there may be ruptures of pregnancy in the right uterine horn. Combined with the physical signs and laboratory examination results, the bleeding due to the rupture of cornual pregnancy was considered. Subsequently, the blood was extracted from the abdominal cavity without coagulation, and the emergency laparotomy was performed immediately. During surgery, the right fundus of the uterus was found near the breach of the uterine horn, and the placenta and amniotic sac were turned out. The right uterine horn was the attachment of the placenta. Although there was some adhesion for the placenta, no obvious residues were observed after artificial dissection, and the base layer was retained. The uterus contracted after the use of strong contractive drugs. The B-Lynch-sutured uterus was conservatively treated, total hysterectomy was avoided, and the reproductive function of this patient was preserved. The patient was pregnant again in 2021 and delivered a child. This case has been approved by the medical ethics committee of our hospital.

For cornual pregnancy indicated by ultrasound or MRI during pregnancy, termination of pregnancy is the first choice. Before surgery, a comprehensive evaluation should be performed, and sufficient blood products should be prepared. Besides, relevant risks shall be explained to patients. If the placenta is delivered completely and there is no severe bleeding, the uterus incision can be quickly sutured to restore the continuity and integrity of its anatomical structure. Meanwhile, local compression hemostasis and intrauterine injection of strong contraction-promoting agents can be performed. In case of continuous bleeding accompanied by uterine contraction and asthenia, the uterine body or ligation of the uterine artery shall be sutured with B-Lynch sutures. It is a simple and effective conservative treatment method to avoid the loss of reproductive function due to hysterectomy.

## 4. Conclusion

To sum up, cornual pregnancy is a rare ectopic pregnancy. With the increased incidence of this disease, appropriate treatment methods should be reasonably selected according to the condition. Drug abortion may induce severe and persistent side effects. The curettage under the guidance of B-ultrasound or under the uterine cavity suction system is characterized by small damage and low costs. Laparoscopic curettage is a safe and effective treatment method. Even if the uterus perforation may occur, it can be repaired in time, and the ectopic focus can be eliminated, thereby reducing the occurrence of persistent ectopic pregnancy. However, this treatment method is relatively expensive. The above treatment methods should be selected under the condition of early diagnosis by B-ultrasound and stable vital signs. In case of uterine ruptures, timely laparotomy is the only option to save the patient life. In order to save lives and obtain the optimal outcome, it is required to select an appropriate surgical method for the intra-abdominal hemorrhage caused by the rupture of cornual pregnancy.

## Author contributions

**Data curation:** Yizhou Fu.

**Supervision:** Guangju Qi.

**Writing – original draft:** Xiaqin Cai, Ke Hong, Yuefang Zhou.
